# Capacitance Enhancement by Incorporation of Functionalised Carbon Nanotubes into Poly(3,4-Ethylenedioxythiophene)/Graphene Oxide Composites

**DOI:** 10.3390/ma13102419

**Published:** 2020-05-25

**Authors:** Anita Cymann, Mirosław Sawczak, Jacek Ryl, Ewa Klugmann-Radziemska, Monika Wilamowska-Zawłocka

**Affiliations:** 1 Department of Energy Conversion and Storage, Faculty of Chemistry, Gdańsk University of Technology, Narutowicza 11/12, 80-233 Gdańsk, Poland; anita.cymann@pg.edu.pl (A.C.); ewa.klugmann-radziemska@pg.edu.pl (E.K.-R.); 2 Institute of Fluid Flow Machinery, Polish Academy of Sciences, Fiszera 14, 80-231 Gdańsk, Poland; mireks@imp.gda.pl; 3 Department of Electrochemistry, Corrosion and Materials Engineering, Faculty of Chemistry, Gdańsk University of Technology, Narutowicza 11/12, 80-233 Gdańsk, Poland; jacek.ryl@pg.edu.pl

**Keywords:** poly(3,4-ethylenedioxytiophene), functionalised carbon nanotubes, graphene oxide, electrodeposition, ternary composites

## Abstract

This paper reports on the role of oxidised carbon nanotubes (oxMWCNTs) present in poly-3,4-ethylenedioxytiophene (PEDOT)/graphene oxide (GOx) composite. The final ternary composites (pEDOT/GOx/oxMWCNTs) are synthesised by an electrodeposition process from the suspension-containing monomer, oxidised carbon nanotubes and graphene oxide. Dissociated functional groups on the surface of graphene oxide play a role of counter-ions for the polymer chains. Detailed physicochemical and electrochemical characterisation of the ternary composites is presented in the paper. The results prove that the presence of oxMWCNTs in the ternary composites doubles the capacitance values compared to the binary ones (450 vs. 270 F cm^−3^ for PEDOT/GOx/oxMWCNTs and PEDOT/GOx, respectively). The amount of carbon nanotubes in the synthesis solution is crucial for physicochemical properties of the composites, their adhesion to the electrode substrate and the electrochemical performance.

## 1. Introduction

In the dynamically developing world of small portable electronic devices, a massive demand for compact and light energy containers arises. Hence, there has been great interest in the improvement of power and energy density of batteries and electrochemical capacitors. A vital issue in all energy storage devices is electrode material. Therefore, the design of new materials and the enhancement of existing ones is crucial for the development of new generation energy storage systems. Composite materials comprising of two or more components drive tremendous attention because they often present superior properties and synergistic effect. The tailoring of composites’ components and optimization of the ratio between them is a demanding process. 

Conducting polymers (CPs) have been investigated as a well-conducting matrix for a wide variety of composite materials for many applications, including catalysis [1,2,3,4,5], sensing [6,7], corrosion protection [8], energy conversion [9,10] and storage [10,11,12,13,14,15,16]. They play the role of a pseudocapacitive component of the electrodes for supercapacitors [10,11,12,13,17,18] as well as the role of a conducting binder for cathode materials for batteries [14,15,16,19]. Poly(3,4-ethylenedioxythiophene) (PEDOT) has drawn broad interest due to its high chemical stability, fast electrochemical kinetics and superb intrinsic conductivity. Conducting polymers exhibit relatively high energy density, but their capacitance decreases over time. In comparison, carbonaceous materials exhibit excellent chemical stability during long-term cycling and high specific power, but they suffer from low energy density. Hence, much effort goes into combining materials that use different charge storage mechanisms to achieve a synergistic effect in improving specific power and specific energy along with preserving high capacitance during charge/discharge.

Conducting polymer/carbon nanomaterial composites have attracted a lot of attention [20,21,22,23,24,25,26,27,28]. Nowadays, ternary composites combining polymers with two types of carbonaceous nanomaterials of different structures and properties are of special interest. Despite the similarities of composites prepared from the same components, many of the described materials exhibit considerable differences in microstructure, physicochemical and electrochemical behaviour [29,30,31,32,33,34,35]. For instance, Mahakul et al. [31] investigated the ternary composites consisted of PEDOT-poly(sodium4-styrenesulfonate) (PSS), reduced graphene oxide (rGOx) and oxidised carbon nanotubes (oxMWCNTs) prepared by the simple mixing of PEDOT/PSS solution with rGOx and different amount of oxMWCNTs. They tried to find an optimal quantity of carbon nanotubes to increase the conductivity of the material. In their study, 7 wt.% content of oxMWCNTs in the composite led to the maximum conductivity of 3804 S cm^−1^. A further increase of oxMWCNTs results in a decrease in conductivity, probably due to the increase of contact resistance arising from the agglomeration of carbon nanotubes at higher concentrations. On the other hand, Islam et al. [30] prepared composites comprised of similar components (PEDOT/PSS, liquid crystalline graphene oxide (LC GO) and multiwalled carbon nanotubes (MWCNTs) that exhibit conductivity of 321, 387 and 269 S cm^−1^ for the composites containing 5 wt.%, 10 wt.% and 15 wt.% of MWCNTs, respectively, which are approximately ten times lower values than for the composites obtained by Mahakul and co-workers [31]. The capacitance value of these composites also depends on the amount of carbon nanotubes, namely 593, 657 and 561 F g^−1^ (in 1 M H_2_SO_4_ at 5 mV s^−1^, three-electrode system) for the composites with 5 wt.%, 10 wt.% and 15 wt.% MWCNTs, respectively [30]. Weng et al. [32] prepared the composite on titanium foil substrate by casting the solution of PEDOT/PSS, GOx and oxMWCNTs and further reducing with ethylene glycol. The composite showed specific capacitance of 255 F g^−1^ in 6 M NaNO_3_ at 100 mV s^−1^ (measured in the three-electrode system). On the other hand, Chen et al. [33] prepared PEDOT hollow spheres (b-PEDOT) with graphene oxide and oxidised multiwalled carbon nanotubes (b-PEDOT/GOx/MWCNTs) composite which showed a maximum capacitance of 225 F g^−1^ at 0.1 A g^−1^ during galvanostatic charge/discharge in the two-electrode system with 5 M KOH serving as the electrolyte. Kim et al. [34], Shen et al. [33] and Hao et al. [35] investigated electrochemical performance of the ternary composites consisting of polyaniline, modified graphene oxide and oxidised multiwalled carbon nanotubes in 1 M H_2_SO_4_ aqueous composite. Despite comprising of the same components, the composites exhibit different capacitance values measured in a three-electrode system in contact with 1 M H_2_SO_4_ electrolyte, namely 440 F g^−1^ (at 1 A g^−1^) [34], 495 F g^−1^ (at 1 A g^−1^) [36] and 696 F g^−1^ (at 20 mV s^−1^) [35].

Studies on conducting polymer-based composites frequently involve materials obtained chemically, which enhances their variability of composition and properties depending on the preparation procedures. We use an electrochemical approach, which is more convenient to control over the synthesis condition (potential, current, time, electrolyte composition, etc.). This results in highly reproducible thin composite films.

In our previous work, we demonstrated a positive effect of the incorporation of functionalised carbon nanotubes (oxidised or nitrogen plasma modified) [37,38] or graphene oxide [39] into the poly(3,4-ethylenedioxythiophene) (pEDOT) matrix. Such binary composites exhibit improved properties compared to their pure components. However, the described composites have some limitations. For instance, when the amount of carbon nanotubes in the pEDOT/oxMWCNTs is too high, it causes the brittleness of the composite and lowers the adhesion to the electrode substrate [37]. On the other hand, pEDOT/GOx composites exhibit significantly increased capacitance values and a nice layered structure due to the presence of graphene oxide sheets. However, if the composite thickness is too high, the diffusion of ions across the GOx planes is hindered, which results in capacitance decrease [39]. Carbon nanotubes (CNTs) are often used as spacers for graphene-based electrodes to prevent graphene sheets from restacking, which enables the full utilization of graphene’s large specific surface area and improves the electrode performance [40,41,42].

Therefore, in this work, we introduce oxidised carbon nanotubes into the pEDOT/GOx composite to improve diffusion perpendicular to the graphene oxide sheets, and in this way, to improve electrochemical properties. Moreover, we describe a systematic study on the influence of synthesis conditions and amount of CNTs on morphology, structure and adhesion to the electrode substrate of the prepared composite films. Additionally, we show the dependence of deposition charge on the composite layer thickness. A crucial aspect not addressed in the literature concerning similar composites is the influence of ageing of the synthesis solution on the morphology and properties of the composite layers.

## 2. Materials and Methods

### 2.1. Chemicals

Monomer 3,4-ethylenedioxythiophene (EDOT), poly(sodium4-styrenesulfonate) (PSS) and multiwalled carbon nanotubes—MWCNTs (≥98% carbon basis, O.D. × I.D. × L 10 nm ± 1 nm × 4.5 nm ± 0.5 nm × 3-~6 µm) were purchased from Sigma–Aldrich (Merck KGaA, Darmstadt, Germany) and used as received. The graphene oxide suspension (6.8 mg/mL) was prepared via modified Hummers method (supplied by Institute of Electronic Materials Technology (ITME), Warsaw, Poland). Inorganics: H_2_SO_4_ (98 wt.%), HNO_3_ (65 wt.%), K_2_SO_4_, (Avantor Performance Materials Poland S.A., Gliwice, Poland) were of analytical grade and used without any further purification. Electrolytes were prepared using deionised water.

### 2.2. Oxidation of MWCNTs

In order to obtain a homogenous aqueous suspension of multiwalled carbon nanotubes, modification of their surface was implemented. Therefore, 1 g of MWCNTs was added to a round-bottomed flask with 120 mL of 65 wt.% nitrate acid and heated in an oil bath to the temperature above the boiling point of nitric acid (130 °C). Then, the mixture of MWCNTs and acid was stirred with a PTFE coated magnetic stirrer for 24 h under reflux conditions. Afterwards, the mixture was cooled down to the room temperature and poured into the beaker with 400 mL of deionised water. Decantation and refilling the beaker with clean deionised water was repeated three times until a stable suspension of modified carbon nanotubes in water was obtained. The oxidised carbon nanotubes were then filtrated under vacuum conditions using glass fibre filter paper (300 µm in thickness, retention 1.6 µm, MN GF – 1, Macherey Nagel) and rinsed with deionised water. PH of the effluent was controlled, and the process was ceased at pH = 7. Afterwards, the filtration cake was carefully collected and dried at 80 °C under vacuum for 24 h.

### 2.3. Electrochemical Preparation of the Ternary Composites pEDOT/GOx/oxMWCNTs

The electrodeposition process was carried out in an aqueous suspension of carbonaceous materials containing EDOT monomer. The synthesis solution was prepared by adding EDOT monomer (0.015 mol dm^−3^) to deionised water (conductivity < 0.06 µS) and further ultrasonicated for 3 min (amplitude 50%, power 25 W) with Ultrasonic Lab Homogeniser (UP200St, LT Scientific, Hielscher). Afterwards, the appropriate amount of oxMWCNTs was added to the solution and homogenised (sonicated) for 5 min until the temperature of the solution reached 50 °C. Finally, graphene oxide suspension was added to the mixture (to the final concentration of GOx equal to 1 mg ml^−1^) and mixed by shaking. Before the deposition of the composite layers, the solution was deaerated with argon for 1 h. During the deaeration and deposition processes, the synthesis solution was stirred (800 rpm). The composite films were deposited in the three-electrode system at a constant potential of 1 V vs. Ag|AgCl|0.1 M KCl on the working electrode (glassy carbon (GC, 2 mm in diameter), platinum plates (area of 1 cm^−2^) or fluorine-doped tin oxide (FTO) coated glass (area of 1 cm^−2^)); Pt mesh served as a counter electrode (see the experimental set-up presented in Figure S1a in the Supplementary Materials—SM). The process was controlled with the deposition charge (0.2–0.8 C cm^−2^), resulting in a controlled thickness of the prepared layers. After deposition, the prepared composite films were washed with deionised water, transferred to the monomer-free electrolyte solution (1.0 M KCl) and reduced electrochemically at −1.0 V vs. ref Ag|AgCl|3 M KCl for 5 min. For comparison, binary composite PEDOT/GOx and pure polymer PEDOT/PSS were prepared by analogical procedure (see details in SM). 

As mentioned in the introduction, carbon nanotubes are supposed to work as a spacer for graphite flakes that create layered structure of PEDOT/GOx composites. Therefore, four different synthesis suspensions were used with various amounts of oxMWCNTs, namely 0.1, 0.3, 0.5 or 1 mg per 1 mL of the solution, to derive the optimal composition and structure of the final ternary composites. The prepared composite layers are marked as PEDOT/GOx/(y)oxMWCNTs, where y corresponds to oxMWCNTs content (in mg mL^−1^) in the synthesis solution.

### 2.4. Characterization Techniques

The morphology and structure of the surface and cross-section of polymer-based composites layers were investigated using scanning electron microscopy (SEM) with a 20 kV beam accelerating voltage with secondary electron-Everhart–Thornley detector (SE-ETD detector) working in high vacuum mode pressure 10^−4^ Pa. To measure the cross-section, the samples were prepared as follows. After the electrodeposition on FTO-coated glass electrode, the composite layers were washed with deionised water and dried in air at ambient temperature. Then, the samples were treated with liquid nitrogen and broken into two pieces. The chemical composition of PEDOT/GOx/oxMWCNTs was examined with an elemental analysis of light atoms (C, N, S, H) using Thermo Scientific Flash 2000 CHNS analyser. The Raman spectra of the composites were recorded using Raman spectrometer (InVia, Renishaw, Wotton-under-Edge, UK) with an argon ion laser (green: 514 nm) within the wavenumber range of 100–4000 cm^−1^. An Escalab 250Xi spectroscope (Thermo-Fischer Scientific, Waltham, MA, USA) with a monochromatic Al Kα source was used to measure the spectra of X-ray photoelectron spectroscopy (XPS). Charge compensation was implemented during the measurements. The spectra were acquired at a constant analyser pass energy of 10 eV with 0.1 eV steps. All the samples were etched with an anion gun before the spectra acquisition. After subtraction of Shirley-type background, XPS spectra were fitted with mixed Gaussian–Lorentzian peaks. All the electrochemical measurements were carried out with AUTOLAB 302N potentiostat/galvanostat (AUTOLAB, Utrecht, The Netherlands) under Nova software (version Nova 2.1.4, Methrom Autolab, Utrecht, Netherlands), and with SP-200 potentiostat/galvanostat (BioLogic, Seyssinet-Pariset, France) under EC-Lab software. Electrode materials were characterised using cyclic voltammetry (CV) at the scan rates of 10–500 mV s^−1^, galvanostatic charge–discharge at the current densities of 0.5–10 A cm^−2^ (GCPL), and electrochemical impedance spectroscopy with the frequency range of 100 kHz–10 mHz (EIS). In the three-electrode system, the active material electrodeposited on GC served as working electrode, whereas Pt mesh and Ag|AgCl|3 M KCl, served as the counter and reference electrodes, respectively (Figure S1b, SM). The two-electrode symmetric systems were assembled in a SWAGELOK^®^ (Swagelok Company, Solon, OH, USA) cell made of polytetrafluoroethylene (PTFE). Electrode material was deposited on glassy carbon electrodes (deposition charge of 800 mC cm^−2^), separated with Whatman^®^ (Maidstone, UK) glass fibre paper which was soaked with an electrolyte (0.5 M K_2_SO_4_) (Figure S1c, SM).

## 3. Results

### 3.1. Physicochemical Characterisation

The elemental analysis was performed to estimate the amount of carbon originating from GOx and oxMWCNTs in the composites (Table S1, SM). Sulphur appears only in the polymer and is not measurable in graphene oxide and carbon nanotubes. The C/S weight ratio in EDOT equals 2.25, while in PEDOT/GOx/(0.1)oxMWCNTs and PEDOT/GOx/(0.5)oxMWCNTs composites is equal to 2.72 and 3.28, respectively. Based on the CHNS analysis results, we estimated 8.2 wt.% of carbon coming from GOx and oxMWCNTs in PEDOT/GOx/(0.1)oxMWCNTs composite, and 15.5 wt.% in PEDOT/GOx/(0.5)oxMWCNTs. This proves that by increasing the amount of carbon nanotubes in the synthesis solution, one may raise the amount of CNTs incorporated in the polymer matrix. 

**Figure 1:**
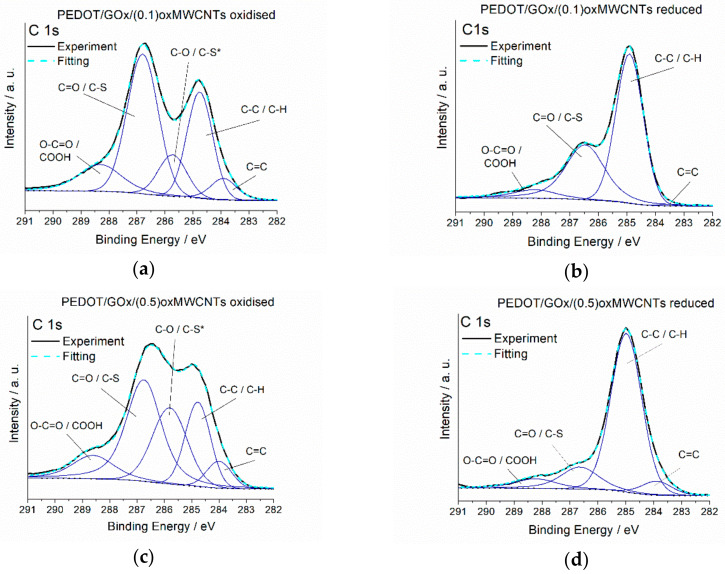
X-ray photoelectron spectroscopy (XPS) C1s spectra recorded for the composites in their oxidised and reduced state; (**a**) poly-3,4-ethylenedioxytiophene/graphene oxide/(0.1)oxidised carbon nanotubes composite in the oxidised state (PEDOT/GOx/(0.1)oxMWCNTs—oxidised) (**b**) PEDOT/GOx/(0.1)oxMWCNTs—reduced, (**c**) PEDOT/GOx/(0.5)oxMWCNTs—oxidised, (**d**) PEDOT/GOx/(0.5)oxMWCNTs—reduced.

To investigate the differences in the oxidation states and binding energies of elements in the studied materials, XPS analyses were performed. XPS spectra were measured for PEDOT/GOx/(0.1)oxMWCNTs and PEDOT/GOx/(0.5)oxMWCNTs in oxidised and reduced forms. During the reduction process, oxygen-rich functional groups on the surface of graphene oxide are irreversibly reduced, as recorded for the pure GOx layer (Figure S2 and Table S2, SM). The same tendency is observed during the electrochemical reduction of the composite layers. Figure 1a–d present XPS spectra of C1s orbitals recorded for the oxidised and reduced composites. The spectra confirm that GOx undergoes partial reduction also in the polymer matrix, as peaks at binding energies in the range of 287–288.5 eV, corresponding to C-O and C=O bonds [43], decrease significantly. The reduction of the ternary composites also leads to an evident increase of atomic content of sp^2^ hybridised C-C bonds: from 17.8 at.% for oxidised to 30 at.% for reduced PEDOT/GOx/(0.1)oxMWCNTs, and from 13.1 at.% for oxidised to 25.1 at.% for reduced PEDOT/GOx/(0.5)oxMWCNTs layer (see Table S2, SM). These results prove the ordering of graphitic layers inside the composite during the reduction process.

**Figure 2:**
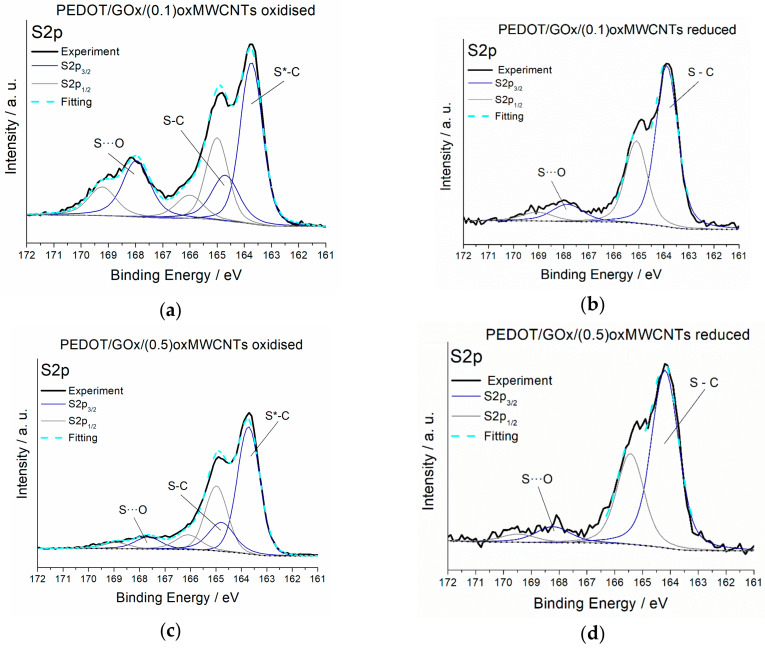
XPS S2p spectra recorded for the composites in their oxidised and reduced state; (**a**) PEDOT/GOx/(0.1)oxMWCNTs—oxidised, (**b**) PEDOT/GOx/(0.1)oxMWCNTs—reduced, (**c**) PEDOT/GOx/(0.5)oxMWCNTs—oxidised, (**d**) PEDOT/GOx/(0.5)oxMWCNTs—reduced.

In our previous work [39], we described the interaction between oxidised sulphur from the thiophene ring (polarons and bipolarons) and negatively charged oxygen groups from the GOx surface in binary composites PEDOT/GOx. This interaction in PEDOT/GOx disappears during electrochemical reduction, probably due to the enlargement of the distance between neutral S from PEDOT and O from GOx. In the ternary composites PEDOT/GOx/oxMWCNTs, such S-O interaction is also observed (doublet at approximately 168 eV, see Figure 2 and Table S2, SM). However, upon reduction, the doublet decreases but does not disappear as it does in PEDOT/GOx. It is likely that the presence of oxMWCNTs in the composite matrix hinders the movement of the components during oxidation and reduction of the polymer chains. Moreover, in the S2p spectra of oxidised PEDOT/GOx/(0.5)oxMWCNTs, the peaks at 168 eV are smaller than in PEDOT/GOx/(0.1)oxMWCNTs, which may indicate that a higher amount of carbon nanotubes in the composite increases the distance between sulphur from PEDOT and oxygen from GOx. These results imply a more rigid structure of the ternary composites compared to the binary ones. Moreover, in the oxidised form of the composites, there are two doublets representing sulphur-carbon bonds at binding energies 163.7 eV (depicted as S*-C) and 164.8 eV (S-C). After the reduction process, only the doublet at higher binding energy is observed, namely 164.5 eV. In the oxidised form of the polymer, a part of the sulphur atoms exist in the form of polarons and bipolarons, whereas in the reduced form, these charge carriers disappear. This may explain the disappearance of one doublet and the increase of intensity of the second one. This is in agreement with C1s spectrum, where one peak representing C-S* bonds disappear in the reduced form of the composites.

**Figure 3:**
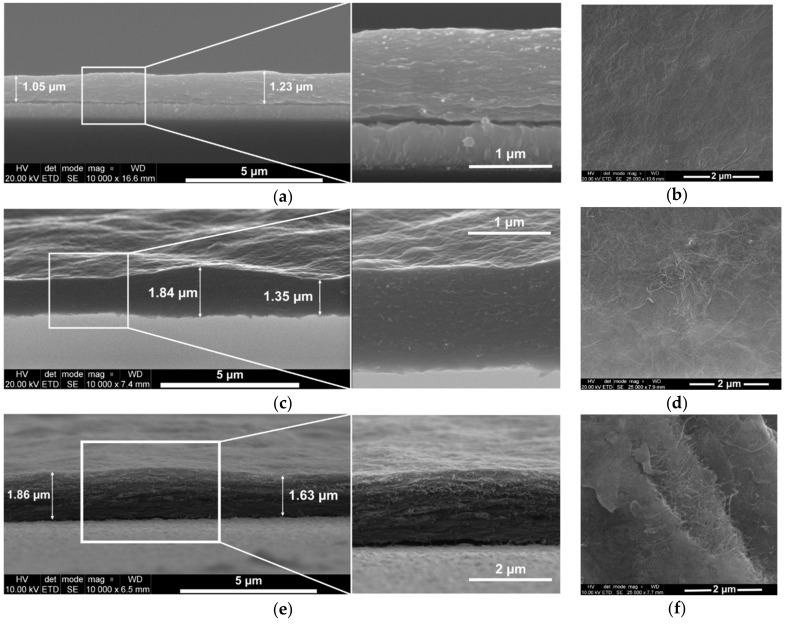
SEM images of the cross-section (**a**,**c**,**e**) and surface (**b**,**d**,**f**) of the composites electrodeposited on fluorine-doped tin oxide (FTO)-coated glass. PEDOT/GOx/(0.1)oxMWCNTs and PEDOT/GOx/(0.5)oxMWCNTs layers deposited with the charge of 0.8 C cm^−2^, PEDOT/GOx/(1)oxMWCNTs layer deposited with 0.2 C cm^−2^.

The influence of the increasing amount of carbon nanotubes in the composites on their morphology and structure was studied by SEM (Figure 3). SEM images of the three composites reveal that more carbon nanotubes are incorporated in the composite matrix from the solution of the higher content of oxMWCNTs (0.1 mg ml^−1^, 0.5 mg ml^−1^ and 1 mg ml^−1^). The amount of CNTs also influence the thickness and the adhesion of the composite layers.

The cross-section pictures of the composite layers show their thickness and morphology. The composites exhibit a layered structure with carbon nanotubes incorporated between GOx layers. Such a layered structure is more pronounced in the composites with higher amount of oxMWCNTs (see also Figure S3, and Figure S4b in SM). The films are quite smooth, but the variation in thickness across the same composite layer rises with the increasing amount of carbon nanotubes in the polymer matrix. The average thicknesses of the composite layers are given in Table 1.

Although the deposition charge of the PEDOT/GOx/(1)oxMWCNTs layer is four times lower than for the other two composites, the layer is thicker. This is caused by a high amount of carbon nanotubes incorporated in the composite matrix, which causes a high variation in thickness (see more SEM images of this composite in Figure S4, SM). Moreover, this composite is the most brittle, and exhibits the most inferior adhesion to the electrode substrate. When PEDOT/GOx/(1)oxMWCNTs is deposited with the charge of 800 mC cm^−2^, the layer breaks and falls off the electrode substrate.

As one can see, the composition of the synthesis solution significantly influences the morphology and structure of the composite layer. Another critical parameter is the age of the prepared solution. The freshly prepared suspension containing GOx, oxMWCNTs and EDOT is colloidal. However, the GOx flakes agglomerate with time in such a complicated mixture. The composite layers prepared from the solution containing agglomerated graphene oxide flakes exhibit radically different morphology. The films are much thicker with substantially irregular porosity and tendency to crack (see Figure S5 in SM). 

**Figure 4:**
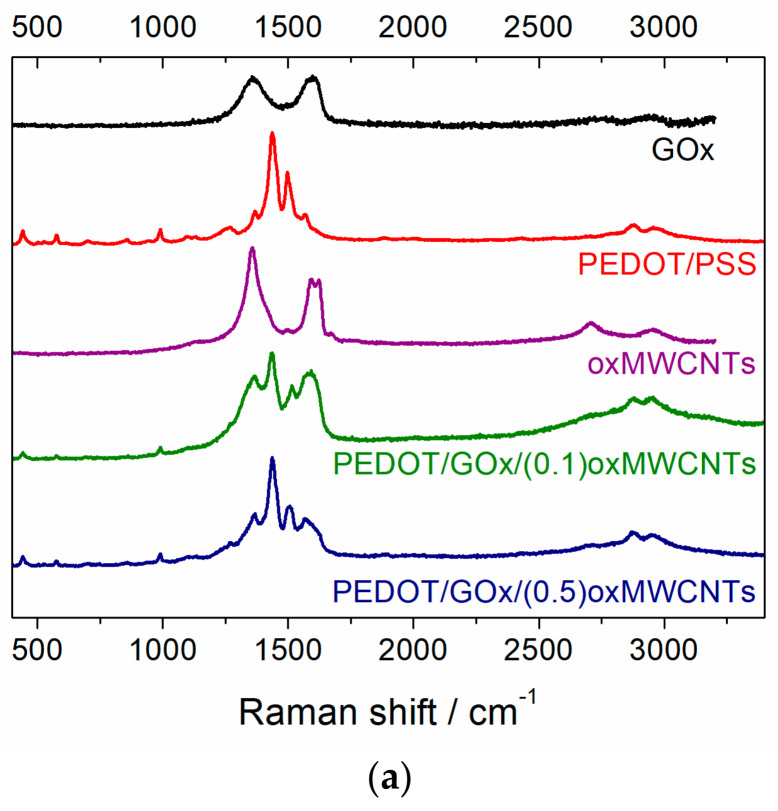

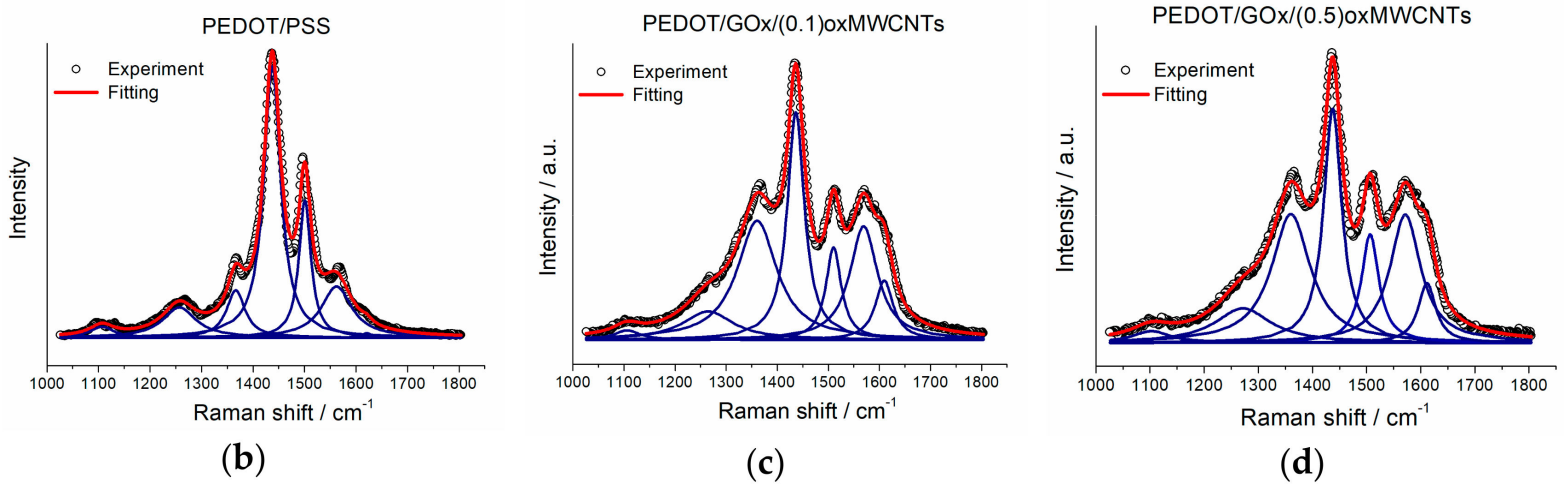
(**a**) Raman spectra of PEDOT-based composites, GOx, oxMWCNTs and PEDOT/poly(sodium4-styrenesulfonate) (PSS); (**b**) deconvoluted Raman spectrum of PEDOT/PSS; (**c**), (**d**) deconvoluted Raman spectra of PEDOT/GOx/(0.1)oxMWCNTs and PEDOT/GOx/(0.5)oxMWCNTs, respectively.

To investigate the carbon structure and to confirm the presence of the individual components in the ternary composites, Raman spectra of oxidised multiwalled carbon nanotubes, graphene oxide, PEDOT/PSS and the composites with different amounts of carbon nanotubes were recorded (Figure 4). Carbonaceous materials exhibit two characteristic bands (D and G) in the first-order Raman spectrum, located at about 1350–1360 and 1570–1590 cm^−1^, respectively. Analysing the position, shape, intensity and width of these bands, one may derive the structure and arrangement of the carbon clusters. In general, D bands represent disorder in the graphitic lattice caused by the presence of sp^3^ carbons and functional groups. On the other hand, the G band originates from the ordered graphite-like sp^2^ carbons [44,45,46,47]. A closer look to the spectrum of oxMWCNTs (Figure S6a in SM) reveals three main bands, namely D at 1358 cm^−1^, G at 1591 cm^−1^ and D’ at 1622 cm^−1^. The presence of a well-pronounced D’ is typical for functionalised CNTs with many defects coming from the presence of surface functional groups [48] and Stone–Wales defects [49]. On the other hand, the Raman spectrum of pure GOx exhibits two broad D and G bands at 1361 cm^−1^ and 1589 cm^−1^, respectively (Figure S6b, SM). Incorporation of these carbonaceous materials in the polymer matrix results in substantial differences between the spectra of the ternary composites and pure polymer (PEDOT/PSS), appearing in a significant broadening of the bands located at 1360 and 1570 cm^−1^ (see Figure 4a and Table S3 in SM). Moreover, in the spectra of the composites, a well-pronounced band at 1610 cm^−1^ is observed (6–7% of the total integrated area), whereas in the PEDOT/PSS spectrum this band is insignificant (0.5% of the total integrated area) and can also be omitted in the fitting procedure without worsening the goodness of fit parameter.

The Raman spectra of PEDOT/GOx/(0.1)oxMWCNTs and PEDOT/GOx/(0.5)oxMWCNTs composites do not demonstrate any significant changes, as they contain the same components. It is likely that the difference in the amount of the carbonaceous materials incorporated in the PEDOT matrix between the investigated ternary composites is too low to be exposed in their Raman spectra.

### 3.2. Electrochemical Characterisation

**Figure 5:**
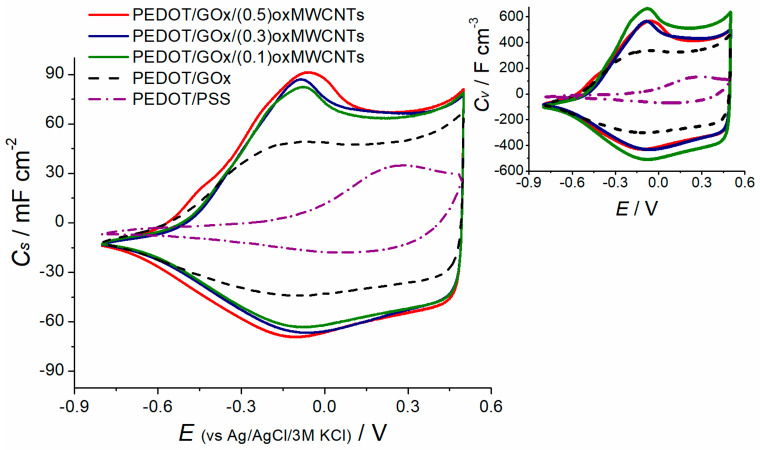
Comparison of the areal capacitance values between ternary composites PEDOT/GOx/oxMWCNTs, binary pEDOT/GOx and pure polymer PEDOT/PSS, scan rate 100 mV s^−1^; Inset: volumetric capacitance values, scan rate 100 mV s^−1^. Recorded in a three-electrode cell in 0.5 M K_2_SO_4_.

Electrochemical measurements in a three-electrode cell configuration were performed to compare the ternary composites PEDOT/GOx/oxMWCNTs with the binary PEDOT/GOx and the pure polymer PEDOT/PSS. Capacitance values based on cyclic voltammetry were calculated according to Equation (1) [50]:

(1) $$C=\frac{I}{v}$$

where: *I*—current [A], *v*—sweep rate [V s^−1^]. The specific capacitance values *C*_s_, presented in Figure 5, were recalculated per area or volume of the active electrode layer.

The CV curves of the ternary composites, regardless of the amount of carbon nanotubes incorporated in their structure, exhibit the same shape and comparable capacitance values. PEDOT/GOx/(0.1)oxMWCNTs shows slightly lower areal capacitance among the ternary composites, but the electrode layer is the thinnest one; therefore it achieves the highest volumetric capacitance values (inset in Figure 5). 

**Figure 6:**
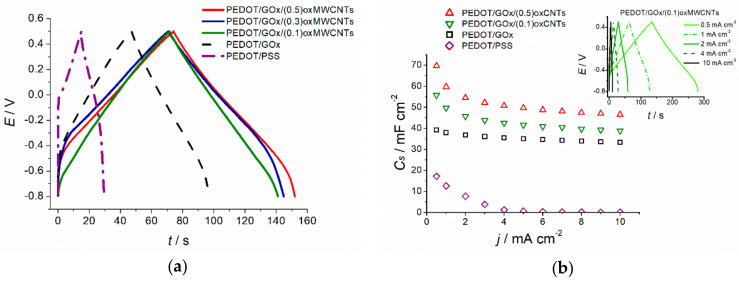
(**a**) Comparison of galvanostatic charge–discharge curves recorded for the investigated materials at 1 mA cm^−2^, (**b**) Areal capacitance values at different current rates; Inset: galvanostatic charge–discharge of PEDOT/GOx/(0.1)oxMWCNTs electrode at different polarisation currents.

Charge–discharge curves of the ternary composites (Figure 6) have a triangular and nearly symmetric shape in the potential range from −0.8 to 0.5 V with no potential drop even at high currents. On the other hand, PEDOT/PSS exhibits significant potential drop (100 mV) upon polarisation with 1 mA cm^−2^ (Figure 1a) and the shortest discharge time, resulting in the lowest capacitance among studied materials. The capacitance values based on the chronopotentiometric measurements were calculated according to Equation (2):

(2) $$C=\frac{I\cdot t}{\Delta E}$$

where: *I*—is the discharge current [A], *t*—is the discharge time [s], Δ*E*—is the potential range [V]. 

The capacitance values decrease upon polarisation with increasing currents, as presented in Figure 6b.

The highest volumetric capacitance value exhibits PEDOT/GOx/(0.1)oxMWCNTs electrode layer (approximately 450 F cm^−3^ at 0.5 mA cm^−2^), which is almost 1.7 times higher than achieved for the binary PEDOT/GOx composite (~270 F cm^−3^ at 0.5 mA cm^−2^) and more than six times higher than for PEDOT/PSS (~70 F cm^−3^ at 0.5 mA cm^−2^). One should bear in mind that the electrode layers of various composites or PEDOT/PSS, electrodeposited with the same charge (here 800 mC cm^−2^), have different thicknesses. Thus, volumetric capacitance may differ even at the same level of areal capacitance. However, the areal capacitance values of the ternary composites still achieves a 1.5 times higher value than the binary composite, and more than three times higher than PEDOT/PSS (55.8, 39 and 17.2 mF cm^−2^ at 0.5 mA cm^−2^ for PEDOT/GOx/(0.1)oxMWCNTs, PEDOT/GOx and PEDOT/PSS, respectively).

**Figure 7:**
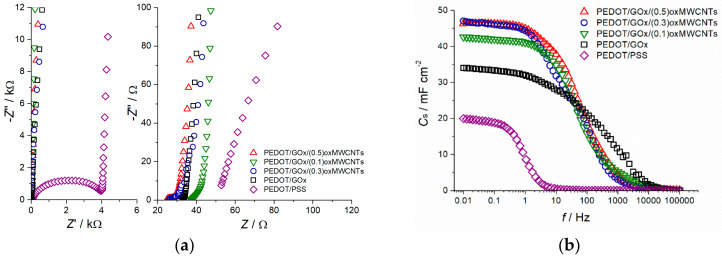
Comparison of (**a**) Nyquist plots, (**b**) specific capacitance vs. frequency, recorded for the investigated materials; electrolyte 0.5 M K_2_SO_4_.

The Nyquist plots of the composites and the pure polymer are displayed in Figure 7a. The ternary PEDOT/GOx/oxMWCNTs and the binary PEDOT/GOx composites exhibit vertical line with no distinct semicircle as observed in PEDOT/PSS layer. In the high frequency region, the impedance of PEDOT/GOx/(0.5)oxMWCNTs layer represents a line shape close to semi-circle of several ohms in diameter, while the other composites show a short line inclined at 40–45 degrees, which is typical for porous electrodes. The Nyquist plot shape of the composites can be described by the finite transmission line model [51,52], which is adequate for representing electron and ion transport in porous conducting polymer electrodes during charge–discharge processes. Vertical line in the low frequency region prove capacitive character of the investigated materials. A significant difference in the shape of the impedance spectra between the composites and PEDOT/PSS may arise not only from different composition but also variation in thickness of the electrode layer. 

The impedance measurements allowed for calculation of the areal capacitance of the investigated materials based on Equation (3):

(3) $$C_{s}=\frac{1}{-Z^{\prime \prime }\cdot 2\cdot \pi \cdot f\cdot s}$$

where: −*Z*ʺ—is the imaginary impedance value [Ω], *f*—is frequency [Hz], *s*—is the geometric area of the electrode layer [cm^2^]. The shape of Bode plots, presented in Figure 7b, prove that the composite layers exhibit considerably better charge propagation than PEDOT/PSS electrode. The capacitance values calculated from the EIS measurements are consistent with the cyclic voltammetry and galvanostatic charge–discharge measurements. 

As we know from the elemental analysis results, the additional carbon coming from both carbonaceous component is equal to 8.2 wt.% and 15.5 wt.% for PEDOT/GOx/(0.1)oxMWCNTs and PEDOT/GOx/(0.5)oxMWCNTs, respectively. Taking into account that the concentration of GOx is from 2 to 10 times higher than the concentration of oxMWCNTs in the synthesis solution, one may roughly estimate the amount of incorporated oxMWCNTs in the range from approximately 1 to a maximum of 7 wt.%. Note that pure oxMWCNTs, due to the moderate specific surface area, show low capacitance values [53,54]. However, they are used as a spacer for GOx flakes incorporated in the PEDOT matrix and a component that provides better porosity, which increases the active surface area available for ions. The presented results prove that the incorporation of a small amount of oxMWCNTs in the PEDOT/GOx matrix leads to a synergistic effect between the components, resulting in a significant enhancement of the capacitance values.

Electrochemical measurements were also performed in the two-electrode symmetric configuration with two identical electrodes. The areal capacitance values based on the galvanostatic charge–discharge measurements were calculated from Equation (4):

(4) $$C_{cell}=\frac{I\cdot t}{U\cdot s}$$

where: *I*—is the discharge current [A], *t*—is the discharge time [s], *U*—is the cell voltage [V], *s*—area of both electrodes [cm^2^].

**Figure 8:**
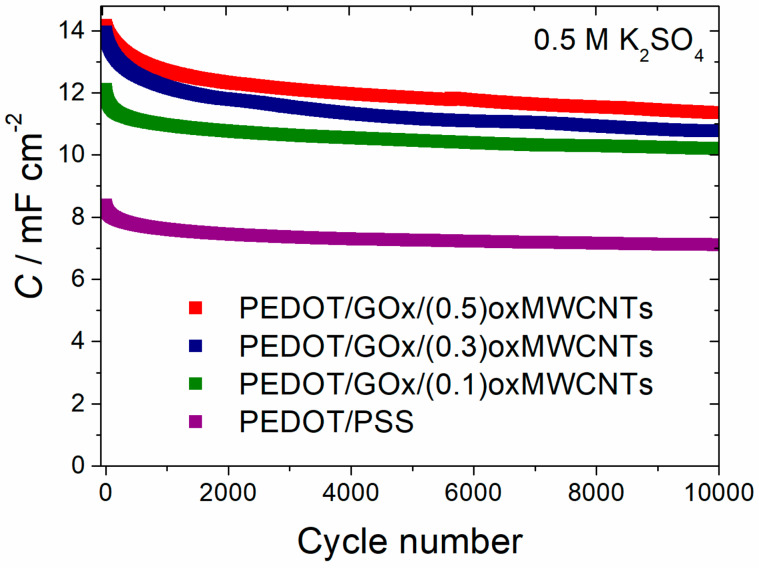
Capacitance as a function of cycle number of the composites recorded for symmetric capacitors with two identical electrodes. Capacitance calculated from galvanostatic charge–discharge (GCPL) measurements: electrolyte 0.5 M K_2_SO_4_; charge/discharge current = 1 mA cm^−2^; cell voltage = 0.5 V.

The highest areal capacitance values were obtained for PEDOT/GOx/(0.5)oxMWCNTs symmetric capacitor, but the best capacitance retention (84% of initial capacitance after 10 000 cycles) was achieved for PEDOT/GOx/(0.1)oxMWCNTs composite (Figure 8). The highest long term stability of the PEDOT/GOx/(0.1)oxMWCNTs electrode layers may be due to its best adhesion to the electrode substrate.

**Figure 9:**
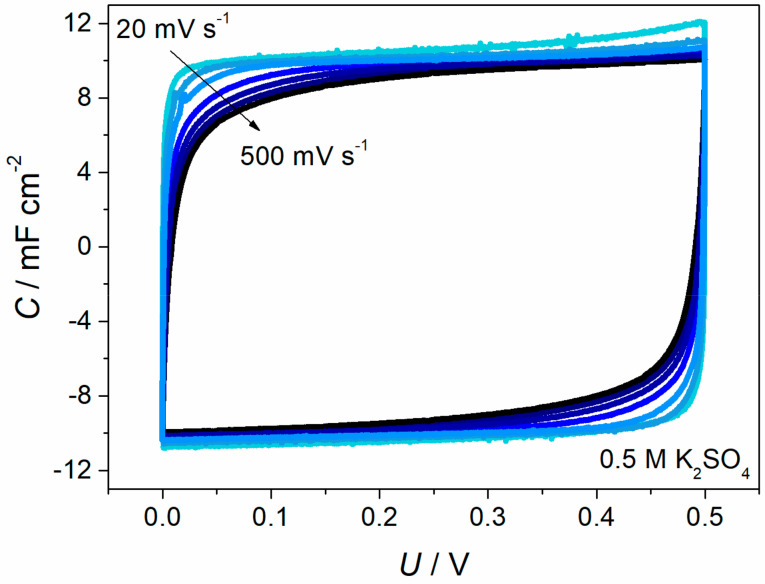
Cyclic voltammetry curves recorded for symmetric supercapacitor with PEDOT/GOx/(0.1)oxMWCNTs composite electrodes at various scan rates 20–500 mV s^−1^.

**Figure 10:**
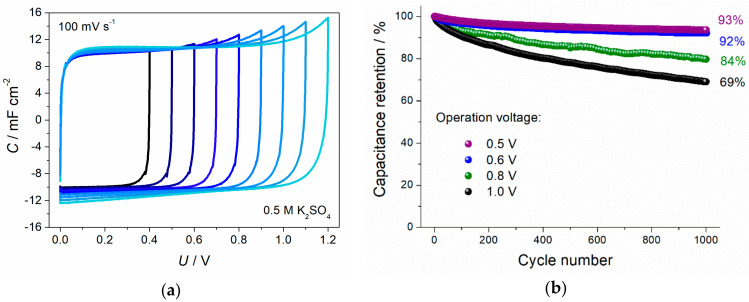
Electrochemical performance of symmetric supercapacitor with PEDOT/GOx/(0.1)oxMWCNTs composite electrodes: (**a**) Cyclic voltammetry curves recorded at different cell voltage; (**b**) Capacitance retention for the symmetric capacitor operating at different voltage.

The CV curves presented in Figure 9 maintain an ideal rectangular shape up to 100 mV s^−1^ with only a slight deviation from the ideal capacitive behaviour up to 500 mV s^−1^, which indicates the fast kinetics of the system and excellent rate capability. CV curves recorded at different cell voltage (Figure 10a) show that the optimum voltage is at 0.6 V or below. The capacitance retention, for such voltage level, remains above 90% after 1000 charge–discharge cycles. Increasing the operation voltage to 0.8 or 1.0 V decreases the capacitance retention to 84% and 69%, respectively (Figure 10b).

The electrochemical results obtained for the ternary PEDOT/GOx/oxMWCNTs composites using three different techniques are consistent and prove high capacitance values, fast kinetics of the electrode processes and low resistance of the electrodeposited layers. These properties make the investigated ternary composites attractive material for high power energy storage devices. Although many similar composites have been reported in the literature, as presented in Table 2, their electrochemical behaviour differs significantly depending on the preparation method and the testing conditions. In addition, slight variations in the structure and proportions of components considerably influence their morphology, physicochemical and electrochemical properties, which, in turn, determine their usefulness as electrodes for energy storage application.

## 4. Conclusions

Ternary composites based on PEDOT, GOx and oxMWCNTs were successfully synthesised by the electrodeposition process. The amount of carbon nanotubes in the suspension for electrosynthesis determines the amount of CNTs incorporated in the composite matrix, which substantially changes the morphology, structure and properties of the electrodeposited composite layers. The addition of the third component, in the form of oxMWCNTs, to the PEDOT/GOx composite leads to a significantly improved electrochemical performance. Carbon nanotubes act as a spacer between graphene oxide layers, as shown in SEM images, enabling better penetration of the electrolyte across the composite layers. This causes the synergistic effect between the components, resulting in considerably enhanced capacitance values, which could not be achieved by the arithmetic sum of the capacities of the single components. However, a too-high amount of the carbon nanotubes in the composite matrix deteriorates the adhesion to the electrode substrate and makes the layers more brittle. Therefore, their amount has to be controlled and their distribution within the layer must be uniform. The electrochemical results achieved for the ternary composites prove their ideal capacitive behaviour with fast kinetics and low resistance. The prolonged electrochemical tests (10,000 charge–discharge cycles) of PEDOT/GOx/(0.1)oxMWCNTs composite in the two-electrode symmetric system indicate that the material is a promising candidate for application in high-power energy storage devices.

## Figures and Tables

**Table 1:** The average thicknesses of the composite layers depending on the amount of oxMWCNTs in the synthesis solution (number in bracket stands for milligrams of oxMWCNTs per 1 mL of the synthesis solution), estimated based of scanning electron microscopy (SEM) images.

Material	Layer Thickness	Deposition Charge
PEDOT/GOx/(0.1)oxMWCNTs	1.24 ± 0.10 µm	800 mC cm^−2^
PEDOT/GOx/(0.3)oxMWCNTs	1.54 ± 0.10 µm	800 mC cm^−2^
PEDOT/GOx/(0.5)oxMWCNTs	1.61 ± 0.19 µm	800 mC cm^−2^
PEDOT/GOx/(1)oxMWCNTs	1.86 ± 0.46 µm	200 mC cm^−2^

**Table 2:** Comparison of capacitance values recorded for ternary composites based on electroactive polymers reported in the literature.

Lp.	Composite	Polymerisation Method	Electrolyte	Capacitance	Δ*E* or *U*	Ref.
1.	PEDOT:PSS/GO^1^/glucose	Chem. ox.^2^ (drop casting)	1 M H_2_SO_4_	19.72 F g^−1^ @ 100 mV s^−1^	−0.4–0.6 V vs. Ag|AgCl	[55]
2.	PEDOT-PSS/GO/CNT	Chem. ox. (film casting on Ti foil)	6 M NaNO_3_	365 F g^−1^ @ 2 mV s^−1^	−1.0–1.0 V vs. Ag|AgCl	[32]
3	b-PEDOT^3^/MWCNTs/rGO^4^	Chem. ox.	5 M KOH	225 F g^−1^ @ 0.1 A g^−1^	−0.2–0.8 V	[33]
4	Free-standing rGO-(10wt%)MWCNT-PEDOT:PSS	Chem. ox. (solution casting)	1 M H_2_SO_4_	761 F cm ^−3^ (657 F g^−1^) @ 5 mV s^−1^	0–0.9 V vs. Ag|AgCl	[30]
5	Activated carbon/ carbon black/ (5wt.%)PEDOT-PSS	Chem. ox. (coating)	1 M TEABF_4_/PC	217 mF cm^−2^ @ 1 mA cm^−2^	0.0–3.0 V	[56]
6	Graphene nanoplatelets/ PEDOT/ eggshell membrane	Chem. ox.	PVA-H_3_PO_4_	39.6 mF cm^–2^ @ 0.3 mA cm^–2^	0–0.8 V	[22]
7	GO/PEDOT-CNTs	EC. dep.^5^ on CNFs, (1 mA cm^−2^; 30 min.; 1.8 C cm^−2^)	1.0 M KCl	95.2 mF cm^−2^ @ 10 mV s^−1^ 100.3 mF cm^−2^ @ 0.5 mA cm^−2^	−0.4–0.6 V vs. SCE	[57]
8	PEDOT/CNT–CG^6^	EC. dep. on graphite paper, (1 mA cm^−2^; 30 min.)	1.0 M KCl	150.6 mF cm^−2^ @ 0.2 mA cm^−2^	−0.5–0.5 V	[29]
9	PEDOT-GO/CNTs	EC. dep. on FTO, (1 mA cm^−2^; 30 min)	1.0 M KCl	99 mF cm^−2^ @ 1 mA cm^−2^	−0.5–0.5 V	[57]
10	PPy/GO/MWCNT	EC. dep. on FTO, (+0.8 V vs. Ag|AgCl; 10 min)	1 M Na_2_SO_4_	358.69 F g^−^^1^ @ 100 mV s^−1^	0–1 V vs. Ag|AgCl	[58]
				~330 F g^−1^ @ 1 A g^−1^	0–1 V	
13	CNF/CNTs/PPy	EC. dep. on CNF, (+0.8 V vs. Ag|AgCl; 200 s)	1 M Na_2_SO_4_	184.13 mF cm^−^^2^ CV, (19.55 mF cm^−^^3^) @ 10 mV s^−1^	0–0.8 V vs. Ag|AgCl	[59]
14	PEDOT/GOx/ (0.1)oxMWCNTs PEDOT/GOx/ (0.3)oxMWCNTs PEDOT/GOx/ (0.5)oxMWCNTs	EC. dep. (1 V vs. Ag|AgCl|0.1 M KCl, 0.8 C cm^−2^)	0.5 M K_2_SO_4_	49.7 mF cm^−2^ @ 1 mA cm^−2^ 56.9 mF cm^−2^ @ 1 mA cm^−2^ 59.8 mF cm^−2^ @ 1 mA cm^−2^	−0.8–0.5 V vs. Ag|AgCl|3M KCl	This work
15	PEDOT/GOx/ (0.5)oxMWCNTs	EC. dep. (1 V vs. Ag|AgCl|0.1 M KCl, 0.8 C cm^−2^)	0.5 M K_2_SO_4_	14.1 mF cm^−2^ @ 1mA cm^−2^ (11.3 mF cm^−2^ after 10,000 cycles)	0–0.8V	This work

^1^ GO—graphene oxide; ^2^ Chem. ox.—chemical oxidation of monomer; ^3^ b-PEDOT—anchored PEDOT hollow spheres; ^4^ rGO—chemically reduced graphene oxide; ^5^ EC. dep.—electrochemical deposition; ^6^ CG—carboxyl graphene.

## Supplementary Materials

The following are available online at https://www.mdpi.com/1996-1944/13/10/2419/s1. Table S1: Elemental analysis results obtained for the composites. Table S2: Results of XPS spectra analysis performer for GOx and the composites in their oxidised (after electrodeposition) and reduced (electrochemically at −1V vs. Ag/AgCl) state. Table S3: Data obtained from curve fitting of the Raman spectra of the investigated materials. Figure S1: Experimental set-up for: (a) electrodeposition process; (b) electrochemical measurements in the three-electrode configuration, (c) electrochemical tests of symmetric capacitor. Figure S2: XPS spectra of C1s orbital recorded for (a) graphene oxide layer, (b) electrochemically reduced graphene oxide layer. Figure S3: SEM image of broken PEDOT/GOx/(0.5)oxMWCNTs composite layer electrodeposited on FTO-coated glass, Figure S4: SEM image of PEDOT/GOx/(1)oxMWCNTs composite layer electrodeposited on FTO-coated glass, deposition charge 200 mC cm^−2^; (a) magnification 10,000, (b) magnification 50,000. Figure S5: SEM pictures of (a) cross-section, (b) surface of PEDOT/GOx/(0.5)oxMWCNTs electrodeposited from the solution containing agglomerated graphene oxide flakes (3 weeks after preparation of the synthesis suspension), deposition charge 800 mC cm^−2^. Figure S6: Deconvoluted Raman spectra of (a) oxMWCNTs, (b) GOx.
