# Supplementary material for: Capacitance Enhancement by Incorporation of Functionalised Carbon Nanotubes into Poly(3,4-Ethylenedioxythiophene)/Graphene Oxide Composites
Source: Materials (Basel). 2020 May 25;13(10):2419. doi: 10.3390/ma13102419 (PMC7287990; doi:10.3390/ma13102419)
Supplement: Supplementary file 1 [file materials-13-02419-s001.pdf]

materials

Supplementary Materials

Capacitance Enhancement by Incorporation of Functionalised Carbon Nanotubes into Poly(3,4-Ethylenedioxythiophene)/Graphene Oxide Composites

Anita Cymann [1], Mirosław Sawczak [2], Jacek Ryl [3], Ewa Klugmann-Radziemska [1] and Monika Wilamowska-Zawłocka [1,*]

[1] Department of Energy Conversion and Storage, Faculty of Chemistry, Gdańsk University of Technology, Narutowicza 11/12, 80-233 Gdańsk, Poland; anita.cymann@pg.edu.pl (A.C.); ewa.klugmann-radziemska@pg.edu.pl (E.K.-R.)

[2] Institute of Fluid Flow Machinery, Polish Academy of Sciences, Fiszera 14, 80-231 Gdańsk, Poland; mireks@imp.gda.pl

[3] Department of Electrochemistry, Corrosion and Materials Engineering, Faculty of Chemistry, Gdańsk University of Technology, Narutowicza 11/12, 80-233 Gdańsk, Poland; jacek.ryl@pg.edu.pl

* Correspondence: monika.wilamowska@pg.edu.pl; Tel.: +48-58-347-24-74

Received: 30 April 2020; Accepted: 22 May 2020; Published: 25 May 2020

Preparation of PEDOT/GOx Composite Layer.

PEDOT/GOx layers were deposited from the solution containing monomer EDOT (0.015 mol dm^{-3}) and graphene oxide (1 mg per 1 ml of the solution). The composite films were deposited in the three-electrode system at a constant potential of 1 V vs Ag|AgCl|0.1 M KCl on the working glassy carbon electrode (GC, 2 mm in diameter), with a deposition charge of 0.8 C cm^{-2}; Pt mesh served as a counter electrode.

Preparation of PEDOT/PSS Layer.

PEDOT/PSS layers were deposited from the solution containing monomer EDOT (0.015 mol dm^{-3}) and sodium polystyrenesulphonate (0.1 mol dm^{-3}). The polymer layers were deposited in the three-electrode system at a constant potential of 1 V vs Ag|AgCl|0.1 M KCl on the working glassy carbon electrode (GC, 2 mm in diameter), with a deposition charge of 0.8 C cm^{-2}; Pt mesh served as a counter electrode.

Table S1. Elemental analysis results obtained for the composites.

	N [wt%]	C [wt%]	H [wt%]	S [wt%]	C/S
pEDOT/GOx/(0.1)ox-MWCNTs	0	47.24	3.07	17.36	2.72
pEDOT/GOx/(0.5)ox-MWCNTs	0	49.52	2.52	15.10	3.28

Table S2. Results of XPS spectra analysis performer for GOx and the composites in their oxidised (after electrodeposition) and reduced (electrochemically at -1V vs. Ag/AgCl) state.

	**GOx oxidised**				**GOx reduced**		
	**BE/eV**	**atomic %**	**assignment**		**BE/eV**	**atomic %**	**assignment**
**O 1s**				**O 1s**	530.45	6.72	O–C=O
	531.81	5.48	O=C/C-O-C		531.7	36.12	O=C / C-O-C
	533.04	25.56	O-C		533.17	3.92	O-C
**C 1s**	284.4	5.95	C=C	**C 1s**	284.27	27.48	C=C
	285.23	21.68	C-C/C-H		285.08	8.57	C-C/C-H
	287.23	32.73	C=O		287.01	12.66	C=O
	288.52	7.08	O-C=O / COOH		288.47	3.07	O-C=O / COOH
	**PEDOT/GOx/(0.1)oxMWCNTs oxidised**				**PEDOT/GOx/(0.1)oxMWCNTs reduced**		
	**BE/eV**	**atomic %**	**assignment**		**BE/eV**	**atomic %**	**assignment**
**O 1s**	531.5	5.45	O=C / O-S	**O 1s**	531.6	9.8	O=C / O-S
	532.7	24.1	O-C / C-OH		533.4	13.09	O-C / C-OH
**C 1s**	283.9	3.92	C=C	**C 1s**	284.1	0.29	C=C
	284.8	17.79	C-C/C-H		285.4	43.63	C-C/C-H
	285.7	7.65	C-O/C-S*				
	286.8	28.32	C=O/C-S		287.0	26.34	C=O/C-S
	288.3	8.68	O-C=O / COOH		288.7	4.59	O-C=O / COOH
**S $2p_{3/2}$**	163.7	1.93	S* -C	**S $2p_{3/2}$**	164.4	1.94	S-C
	164.7	0.74	S-C				
	168.0	0.91	S···O		168.3	0.31	S···O
	**PEDOT/GOx/(0.5)oxMWCNTs oxidised**				**PEDOT/GOx/(0.5)oxMWCNTs reduced**		
	**BE/eV**	**atomic %**	**assignment**		**BE/eV**	**atomic %**	**assignment**
**O 1s**	531.48	5.19	O=C / O-S	**O 1s**	531.3	13.9	O=C / O-S
	532.89	22.78	O- C / C- OH		533.6	5.37	O-C / C-OH
**C 1s**	284	4.16	C=C	**C 1s**	284.2	5.1	C=C
	284.78	13.08	C-C/C-H		285.3	57.32	C-C/C-H
	285.8	16.53	C-O/C-S*				
	286.76	25.26	C=O/C-S		286.9	11.18	C=O/C-S
	288.61	6.98	O-C=O / COOH		288.5	5.43	O-C=O / COOH
**S $2p_{3/2}$**	163.7	4.23	S*-C	**S $2p_{3/2}$**	164.5	1.52	S-C
	164.8	1.26	S-C				
	167.7	0.53	S···O		168.5	0.19	S···O

Table S3. Data obtained from curve fitting of the Raman spectra of the investigated materials.

	Peak position/cm^{-1}	% of the total integrated area	FWHM
**oxMWCNTs**	1358.4	66.94	79.14
	1591.4	24.48	43.96
	1622.4	8.58	19.98
**GOx**	1361.1	59.17	138.31
	1588.7	40.83	89.28
**PEDOT/PSS**	1106.9	2.42	55.59
	1257.2	9.84	78.57
	1366.5	8.88	43.51
	1437.3	46.18	39.37
	1500.7	16.83	28.17
	1562.2	15.38	72.09
	1621.0	0.47	24.61
**PEDOT/GOx/(0.1)oxMWCNTs**	1106.0	1.46	62.63
	1264.6	8.53	119.75
	1360.7	29.16	94.30
	1436.6	25.16	40.65
	1510.3	9.19	36.47
	1569.2	19.72	65.86
	1609.6	6.78	42.57
**PEDOT/GOx/(0.5)oxMWCNTs**	1103.0	2.14	86.10
	1271.6	9.90	125.42
	1360.2	26.78	86.86
	1437.3	23.56	40.33
	1506.5	10.62	39.25
	1571.6	21.24	68.82
	1611.6	5.77	39.02

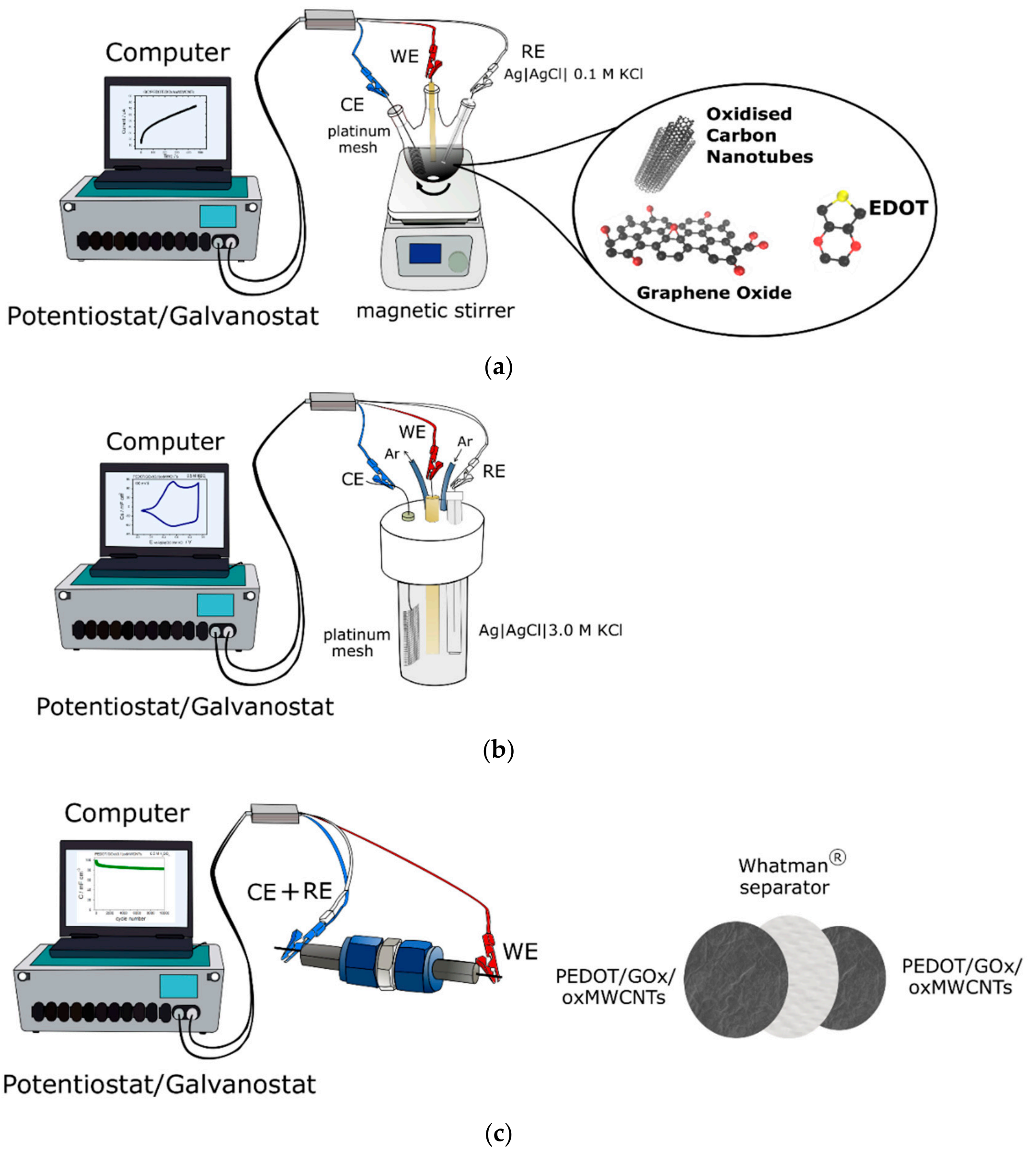

Figure S1. Experimental set-up for: (**a**) electrodeposition process; (**b**) electrochemical measurements in the three-electrode configuration, (**c**) electrochemical tests of symmetric capacitor.

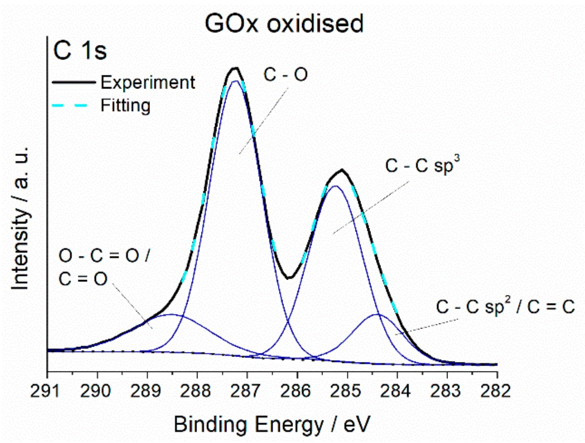

(**a**)

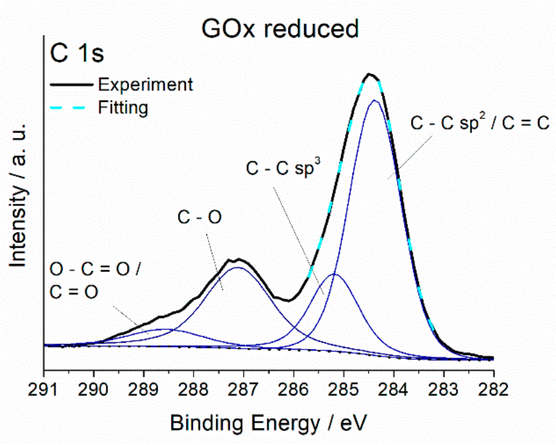

(**b**)

Figure S2. XPS spectra of C1s orbital recorded for (**a**) graphene oxide layer, (**b**) electrochemically reduced graphene oxide layer.

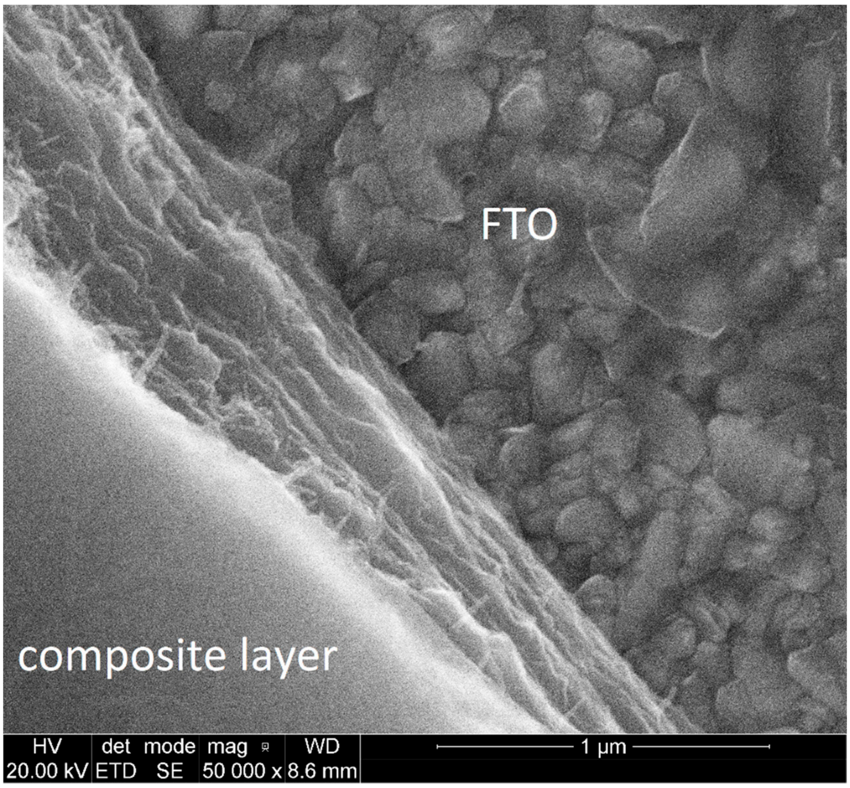

Figure S3. SEM image of broken PEDOT/GOx/(0.5)oxMWCNTs composite layer electrodeposited on FTO-coated glass.

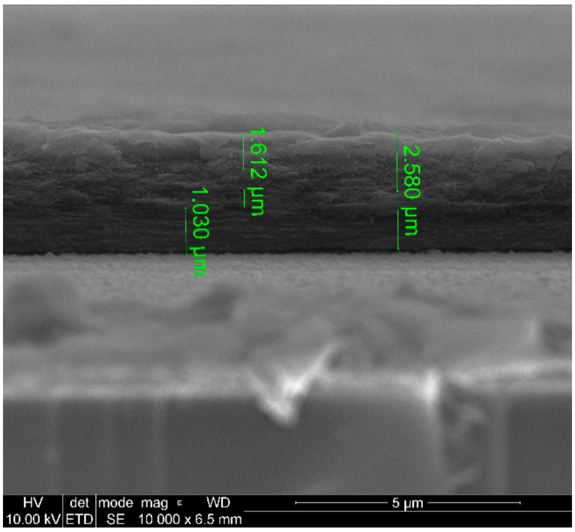

(a)

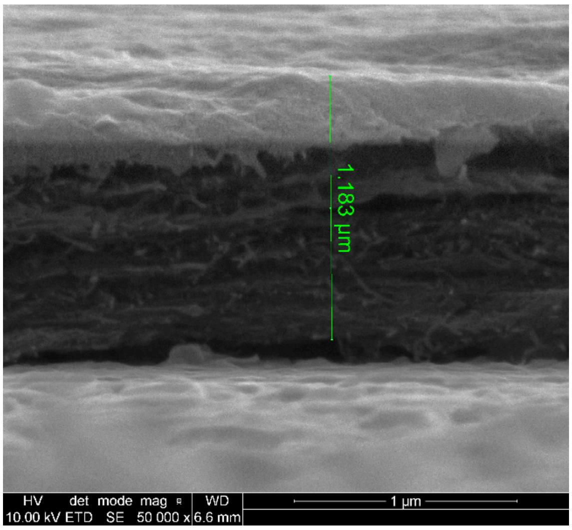

(b)

Figure S4. SEM image of PEDOT/GOx/(1)oxMWCNTs composite layer electrodeposited on FTO-coated glass, deposition charge 200 mC cm^{-2}; (**a**) magnification 10,000, (**b**) magnification 50,000.

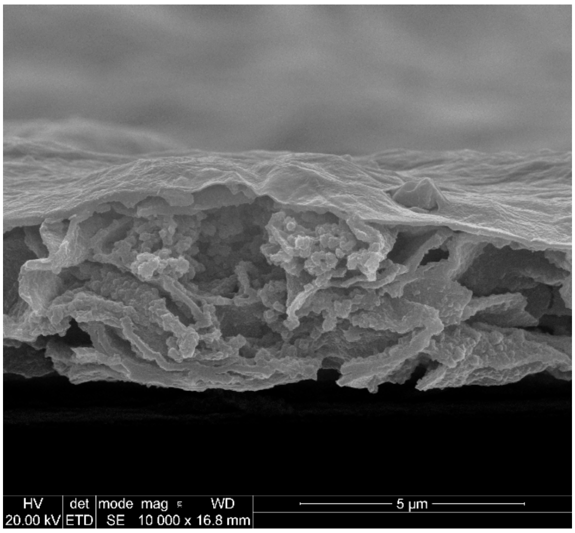

(a)

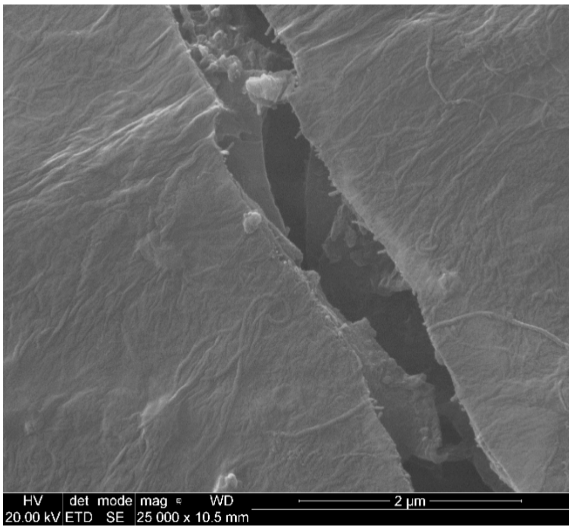

(b)

Figure S5. SEM pictures of (**a**) cross-section, (**b**) surface of PEDOT/GOx/(0.5)oxMWCNTs electrodeposited from the solution containing agglomerated graphene oxide flakes (3 weeks after preparation of the synthesis suspension), deposition charge 800 mC cm^{-2}.

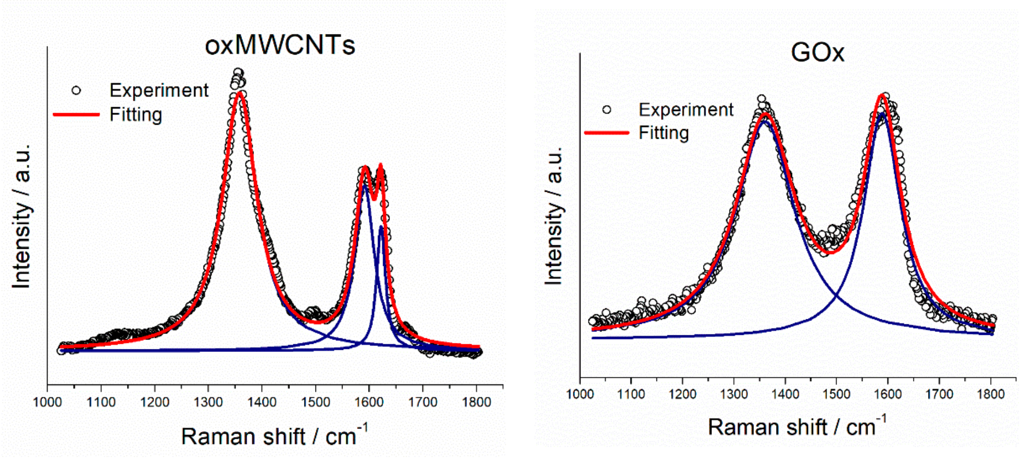

(**a**) (**b**)

Figure S6. Deconvoluted Raman spectra of (**a**) oxMWCNTs, (**b**) GOx.

© 2020 by the authors. Licensee MDPI, Basel, Switzerland. This article is an open access article distributed under the terms and conditions of the Creative Commons Attribution (CC BY) license (http://creativecommons.org/licenses/by/4.0/).
